# Polymer-Supported Poly(Ethylene Glycol) as a Phase-Transfer Catalyst for Cross-Aldol Condensation of Isobutyroaldehyde and Formaldehyde

**DOI:** 10.3390/molecules27196459

**Published:** 2022-09-30

**Authors:** Agnieszka Siewniak, Edyta Monasterska, Ewa Pankalla, Anna Chrobok

**Affiliations:** 1 Department of Chemical Organic Technology and Petrochemistry, Faculty of Chemistry, Silesian University of Technology, Krzywoustego 4, 44-100 Gliwice, Poland; 2 Grupa Azoty Zakłady Azotowe Kędzierzyn, S.A., Mostowa 30A, 47-220 Kędzierzyn-Koźle, Poland; 3 Department of Chemical Organic Technology and Petrochemistry, PhD School, Silesian University of Technology, Akademicka 2A, 44-100 Gliwice, Poland

**Keywords:** hydroxypivaldehyde, cross-aldol condensation, isobutyraldehyde, formaldehyde, phase-transfer catalyst, poly(ethylene glycol)

## Abstract

Immobilized poly(ethylene glycol) (PEG 600-PS) was used as an effective phase-transfer catalyst for the synthesis of hydroxypivaldehyde from isobutyraldehyde (IBA) and formaldehyde in the presence of an inorganic base. Studies on the influence of the parameters on the course of the reaction in a batch reactor showed that the use of the PEG 600-PS catalyst allowed one to obtain HPA with high efficiency (IBA conversion >96%, selectivity >98%) in a relatively short time and under mild conditions (2 h, 40 °C). The developed method enables easy separation of the post-reaction mixture by simple phase separation, and the immobilized catalyst can be separated by filtration and then used five times without a loss in its activity. The high activity and stability of the catalyst was also confirmed in a test carried out in a flow reactor.

## 1. Introduction

**Scheme 1:**
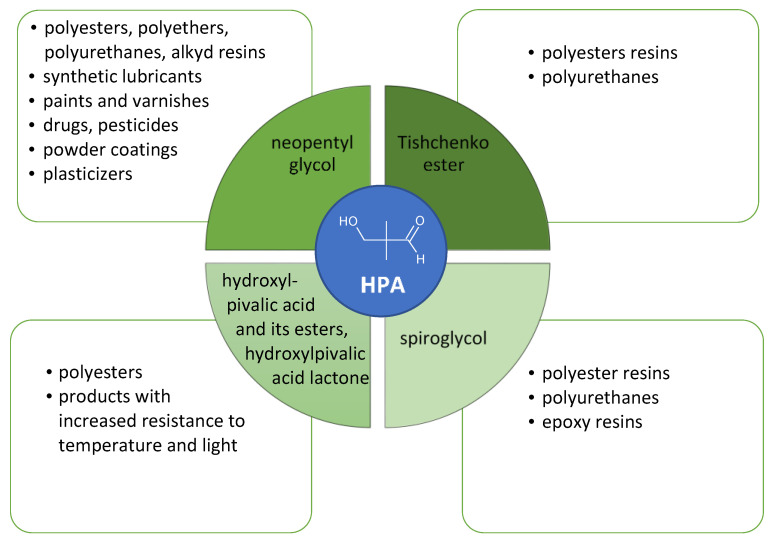
Applications of hydroxypivaldehyde [1,2,3,4,5,6].

The demand for hydroxypivalaldehyde (HPA) continues to grow as it is a valuable intermediate for the production of many compounds of industrial importance, such as additives improving the quality of plastics, and in the production of paints, varnishes and lubricants (Scheme 1) [1,2,3,4,5]. These compounds include: neopentyl glycol (2,2-dimethyl-1,3-propanodiol, NPG), which is obtained mainly by hydrogenation of hydroxypivaldehyde [3,6,7]; neopentyl glycol monohydroxypivalate, so-called Tishchenko ester (ET), obtained by HPA disproportionation (Tishchenko reaction) [5,6]; and spiroglycol (3,9-bis(1,1-dimethyl-2-hydroxyethyl)-2,4,8,10-tetraoxaspiro[5.5]undecane) synthesized from hydroxypivaldehyde and pentaerythritol [8,9,10]. Other compounds obtained from HPA are, among others, hydroxypivalic acid and its esters, hydroxypivalic acid lactone.

On an industrial scale, hydroxypivaldehyde is synthesized mainly by cross-aldol condensation of isobutyraldehyde (IBA) with formaldehyde (FA). The reaction is classically catalyzed by inorganic bases [11,12,13] or tertiary amines [14,15,16]. However, many of these catalytic systems suffer from disadvantages such as large amounts of bases, resulting in a significant amount of wastewater that must be neutralized and utilized, corrosion of the apparatus caused by the use of concentrated bases, and side reactions such as the Cannizzaro or the Tishchenko reactions. Therefore, recent studies have focused on the use of heterogeneous catalysts such as ion-exchange resins [7,17], perovskites [18], metal nitrides [18], and layered double hydroxides [19,20,21] as catalysts.

Phase-transfer catalysis (PTC) is an effective technique for conducting reactions in two-phase system [22,23,24,25]. The PTC market has been expanding significantly and is dedicated to the chemical industry due to its numerous advantages, including: the possibility of the selection of green solvents or even eliminating an organic solvent; the possibility of replacing toxic, expensive and troublesome reagents; and mild process conditions. This contributes to lowering the process costs and reducing the amount of waste. Moreover, the processes carried out under PTC conditions occur at high rates, high yields and high selectivity. Therefore, PTC is an ideal tool for the adoption of green chemistry principles into organic synthesis.

There are only a few examples of the use of PTC in the synthesis of hydroxypivaldehyde in the literature. In addition to the heterogeneous basic catalyst in the form of hydrated-layered double hydroxides, a cocatalyst cetyltrimethylammonium bromide (CTAB) was used, which acted as a phase-transfer catalyst, facilitating the migration of IBA from the organic phase to the aqueous phase [19,20,21]. Hashmi reported benzyltrimethylammonium hydroxide, a basic phase-transfer catalyst which provided excellent results for hydroxypivaldehyde [26]. However, such PT catalysts are soluble in the reaction medium. Typical methods for separating the catalyst from reaction mixture, such as distillation or extraction, constitute an additionally costly and energy-consuming operation in the process. Therefore, in recent years, great emphasis has been placed on searching for techniques enabling easy separation of the catalyst. In particular, there is an interest in the heterogenization of catalysts on insoluble supports [27].

The immobilization of the homogeneous catalyst on a solid support offers many advantages. Similarly to heterogeneous catalysts, the immobilized catalysts can be easily separated from the post-reaction mixture, e.g., by filtration, and reused to subsequent processes [27,28,29]. Moreover, the immobilization of the catalyst on the support makes it possible to use it in continuous systems. The disadvantages of such a catalyst may be its lower activity and usually higher price compared to the homogeneous counterpart. However, the ease of separation of the post-reaction mixture and multiple recycling of the catalyst can largely compensate for the expenses related to the immobilization of the catalyst.

Here, we report the first use of immobilized poly(ethylene glycol) as an effective phase-transfer catalyst for condensation of isobutyraldehyde with formaldehyde and show that it can be recycled without changing its activity.

## 2. Results and Discussion

### 2.1. Batch System

**Scheme 2:**
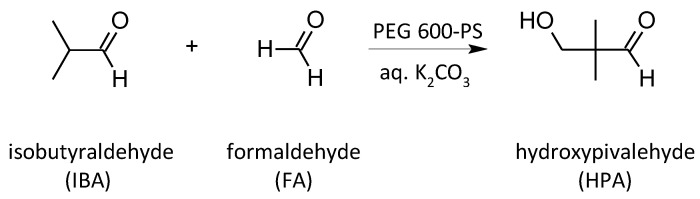
Synthesis of hydroxypivaldehyde from isobutyroaldehyde and formaldehyde.

**Scheme 3:**
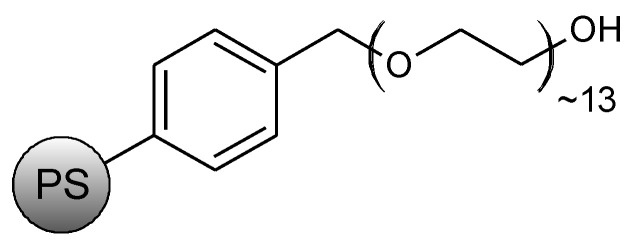
PEG 600-PS catalyst used in the study.

The studies on cross-condensation of isobutyraldehyde with formaldehyde under phase-transfer catalysis conditions were carried out in the presence of immobilized poly(ethylene glycol) (PEG) as a phase-transfer catalyst and in the presence of K_2_CO_3_ (Scheme 2). The process took place in a two-phase system, in which isobutyraldehyde formed the organic phase, and the inorganic phase was composed of an aqueous solution of formaldehyde and an aqueous solution of a base. Poly(ethylene glycol) of a molecular weight of 600 g/mol, immobilized on a polymer support, which was polystyrene cross-linked with 1% divinylbenzene (PEG 600-PS), was used as a PT catalyst to facilitate the contact of the separate phases (Scheme 3). The number of active groups of the catalyst on the support was 0.35 mmol/g OH.

In order to check whether the reactants’ diffusion to the active sites of the catalyst has an influence on the course of the reaction in a heterogeneous system, the effect of stirring on the HPA synthesis was determined. The other reaction conditions remained unchanged (IBA:FA 1.2:1; 40 wt.% K_2_CO_3_, 40 °C, 2 h).

**Figure 1:**
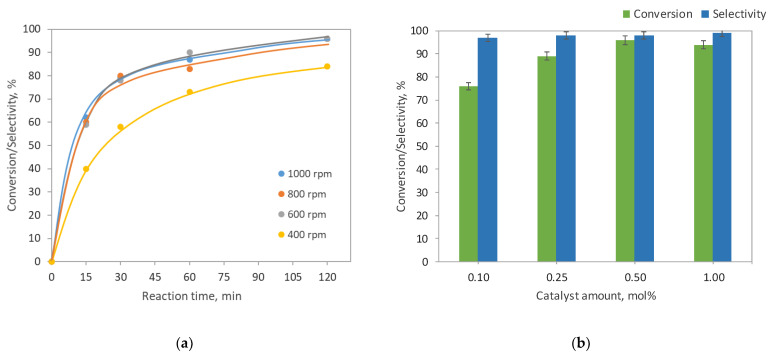
The effect of (**a**) agitation speed; (**b**) catalyst amount on hydroxypivaldehyde synthesis. Reaction conditions: (**a**) IBA:FA molar ratio 1.2:1; 40 wt.% K_2_CO_3_; PEG 600-PS, 0.5 mol%; 40 °C; 2 h; (**b**) IBA:FA molar ratio 1.2:1; 40 wt.% K_2_CO_3_; PEG 600-PS; 600 rpm; 40 °C; 2 h. Each experiment was performed three times.

The results presented in Figure 1a indicate that, above 600 rpm, the process rate was not limited by mass transport. Therefore, the subsequent experiments were conducted at a speed of 600 rpm.

Figure 1b shows the influence of the amount of the immobilized PEG 600-PS catalyst on the course of HPA synthesis. The catalyst was used in an amount ranging from 0.1 to 1.0 mol% with respect to the FA, with the other reaction conditions being maintained (IBA: FA 1.2:1; 40 wt.% K_2_CO_3_, 600 rpm, 40 °C). The conversion of isobutyraldehyde increased with the increase in the amount of catalyst in the range from 0.1 mol% to 0.5 mol%. However, above this value, the amount of catalyst did not have a significant influence on the course of the reaction. Further tests were carried out using 0.5 mol% of the catalyst in relation to FA.

**Figure 2:**
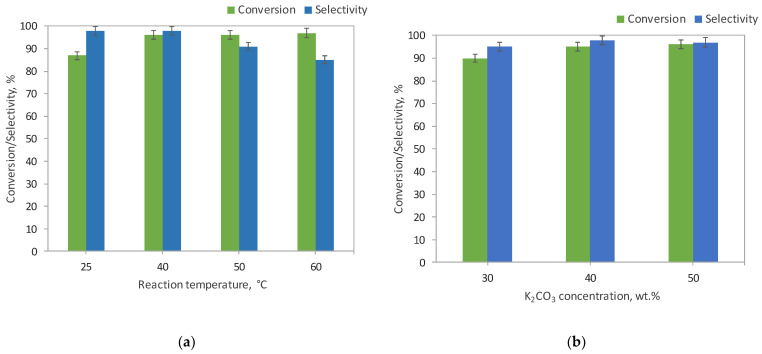
The effect of (**a**) temperature; (**b**) K_2_CO_3_ concentration, on hydroxypivaldehyde synthesis. Reaction conditions: (**a**) 40 wt.% K_2_CO_3_; PEG 600-PS, 0.5 mol%; 600 rpm; 2 h; (**b**) K_2_CO_3_; PEG 600-PS, 0.5 mol%; 40 °C; 600 rpm; 2 h. Each experiment was performed three times.

As the temperature increased, the conversion of IBA increased (Figure 2a), but at the same time, so did the number of by-products formed: neopentyl glycol and Tishchenko ester also increased, which was confirmed by GC-MS analysis. On the basis of the obtained results, it can be concluded that the most-preferred temperature for the HPA synthesis in the presence of the immobilized PEG 600-PS catalyst was 40 °C.

The influence of the concentration of potassium carbonate solution on the conversion of isobutyraldehyde was examined. Based on the results shown in Figure 2b, it can be concluded that the concentration of the base above 40% did not have a significant effect on the course of the reaction; therefore, 40 wt.% aqueous K_2_CO_3_ solution was used for further research.

The impact of changing the reagent molar ratios (isobutyraldehyde in relation to formaldehyde) was also investigated. Butyraldehyde was used in excess to formaldehyde in the range of 1.1:1 to 1.3:1 (mol/mol). The remaining reaction conditions were the same: 40 wt.% K_2_CO_3_, 0.5 mol% PEG 600-PS, temperature 40 °C, 600 rpm. Table 1 shows that, as the IBA:FA ratio was increased, the conversion of IBA practically remained unchanged. However, at the IBA:FA molar ratio of 1:1, the selectivity of the process decreased.

**Figure 3:**
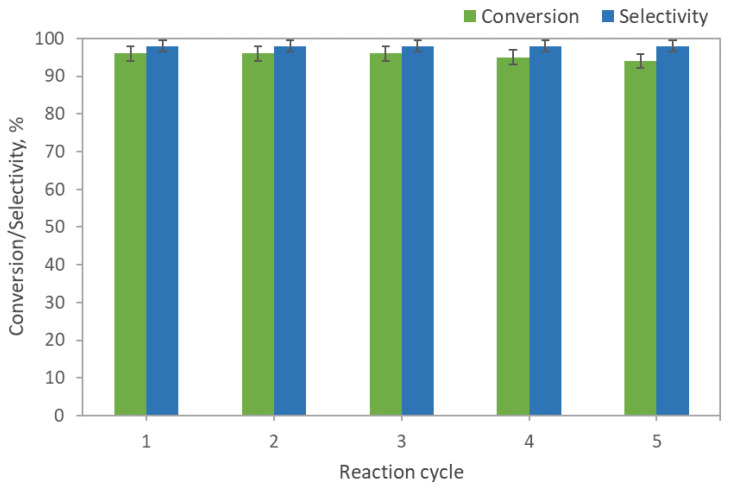
Catalyst recycling tests. Reaction conditions: IBA:FA molar ratio 1.2:1; 40 wt.% K_2_CO_3;_ PEG 600-PS, 0.5 mol%; 600 rpm; 40 °C, 2 h. Each experiment was performed three times.

The major advantage of a heterogeneous catalyst is that it can easily be removed from the reaction mixture. Therefore, the possibility for reuse of the immobilized catalyst was tested. For this purpose, after the completed reaction, the catalyst was separated from the reaction mixture by filtration. The catalyst was then washed with diethyl ether (2 × 10 mL), ethanol (2 × 10 mL) and water (2 × 10 mL). After drying, the catalyst was reused in a subsequent process. The results are shown in Figure 3.

The obtained results confirm the possibility of multiple uses for the immobilized catalyst in the HPA synthesis, without a significant decrease in activity. Moreover, the catalyst is easily separated from the reaction product by filtration, which in turn improves the economy of the process.

### 2.2. Test in a Flow Reactor

**Scheme 4:**
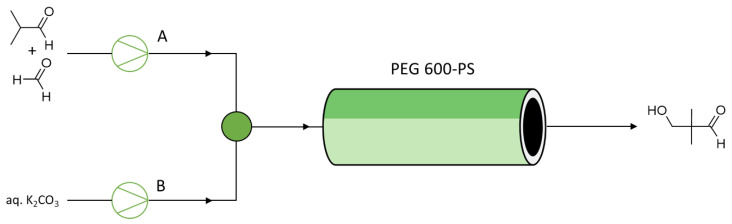
HPA synthesis in a flow reactor. Reaction conditions: IBA:FA molar ratio 1.2:1; 40 wt.% K_2_CO_3;_ PEG 600-PS, 0.5 mol%; 600 rpm; 40 °C, 2 h.

**Figure 4:**
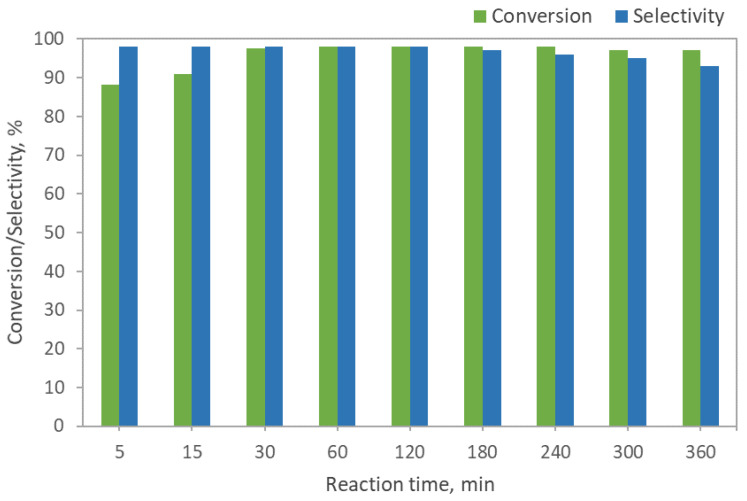
Hydroxypivaldehyde synthesis in a flow reactor. Reaction conditions: 40 wt.% K_2_CO_3_; PEG 600-PS, 0.5 mol%; 40 °C; 600 rpm; 2 h. Reaction time was the sampling time after exceeding the residence time. Flow rate of pump A: 0.01 mL/min; flow rate of pump B: 0.02 mL/min; total flow rate: 0.03 mL/min. Residence time: 50 min.

To prove the effectiveness of developed method, an attempt to synthesize HPA in a flow reactor was undertaken (Scheme 4). The reactor was equipped with two pressure pumps connected to the standard T-mixer. The first pump pumped a stream composed of an aqueous solution of formaldehyde and isobutyraldehyde, and to ensure homogeneity of this stream, the addition of a solvent (isopropanol) was necessary. The second pump was used to deliver an aqueous solution of potassium carbonate to the reactor. The glass column reactor was charged with the PEG 600-PS catalyst (0.35 g). Polymer supports can swell when exposed to solvent, and increased swelling facilitates the diffusion of the reactants to the active centers that are inside of the catalyst. Therefore, in order to establish the exact volume of the reactor, isopropanol was passed through the catalyst bed (Figure S2). The exact volume of the reactor was determined from the volume difference between the empty reactor and the reactor filled with the swollen catalyst and was equal to 1.5 mL (Equation (S1)). The reaction was carried out under the favorable reaction conditions determined during the tests in the batch reactor (IBA:FA molar ratio 1.2:1; 40 wt.% K_2_CO_3;_; 40 °C). Results are shown in Figure 4. During the 6 h reaction, the IBA conversion remained the same without loss of selectivity, which confirms the usefulness of the developed method.

## 3. Materials and Methods

### 3.1. Chemicals

All applied organic reagents and solvents were of reagent-grade purity. Poly(ethylene glycol) 600 bound to polystyrene (1% DVB, 0.35 mmol/g OH loading), formaldehyde (37 wt.% in H_2_O, containing 10–15% methanol as stabilizer to prevent polymerization), and decane anhydrous (≥99%) were purchased from Sigma-Aldrich (Steinheim, Germany), and diethyl ether EMPLURA^®^ was purchased from Merck (Darmstadt, Germany). Isobutyraldehyde was provided by Grupa Azoty Zakłady Azotowe Kędzierzyn, S.A (Kędzierzyn-Koźle, Poland).

### 3.2. Apparatus

Analysis of the samples from reaction mixture was performed on a Shimadzu GC-2010 Plus gas chromatograph (Shimadzu Corp., Kyoto, Japan) equipped with a flame ionization detector (FID) and a Zebron SPB5TM capillary column: 30 m × 0.25 mm × 0.25 mm (Phenomenex, Torrance, CA, USA). The following temperature program was used: 40 °C for 2 min then an increase to 100 °C with rate 10 °C/min; 150 °C with rate 40 °C/min and held for 10 min (Figure S2).

Qualitative GC-MS analysis of the obtained mixtures was performed using a gas chromatography Agilent Technologies GC 7890A (Agilent Technologies, Santa Clara, CA, USA) coupled with a mass spectrometer Agilent Technologies MS 5975C MSD, Triple-AxisDetector (Agilent Technologies, Santa Clara, CA, USA) equipped with a HP-5 MS column (Agilent Technologies, Santa Clara, CA, USA) with dimensions of 30 m × 0.25 mm × 0.25 μm, using helium as s carrier gas (Figure S4). NMR spectroscopy was performed on Varian NMR system: ^1^H NMR spectra at 600 MHz and ^13^C NMR at 151 MHz (Figure S3) [30].

### 3.3. Experimental Procedure

#### 3.3.1. A Batch Procedure

To a round-bottom flask equipped with a reflux condenser formaldehyde (5 mmol) as a 37% aqueous solution, cooled isobutyraldehyde (5–6 mmol) and poly(ethylene glycol) 600 bound to polystyrene (0.05 mmol) were added. The reaction mixture was stirred with a magnetic stirrer. Then, aqueous solution of K_2_CO_3_ was added dropwise to the flask. After that, the process was carried out at given temperature. After completion of the reaction, the catalyst was filtered off and phases were separated. The aqueous layer was washed three times with diethyl ether. The combined organic phase was then washed with water and dried over anhydrous MgSO_4_. A sample from organic phase was analyzed by gas chromatography. A decane was used as an internal standard.

#### 3.3.2. A Flow Procedure

The HPA synthesis test in a continuous mode was performed in a Syrris Asia flow column equipped with a Syrris Asia module consisting of two syringe pumps, a Syrris Asia heater and a Syrris Asia pressure controller (Figure S1). The Omnifit glass reactor (dimensions: 6.6 × 100 mm) placed in the thermostat was filled with a catalyst through which isopropanol was passed through for 30 min to swell the catalyst bed. Then, a mixture of isobutyraldehyde and formaldehyde in isopropanol was pumped by one pump and the potassium carbonate solution by the other one. The two streams were mixed in a standard T-mixer connected to the reactor via tubing. A back-pressure controller installed at the end of the tube exiting the reactor was set to 2 bar. After a residence time, samples were taken at specified times and analyzed by GC.

## 4. Conclusions

An efficient method for the synthesis of hydroxypivalaldehyde by cross-aldol condensation of isobutyraldehyde and formaldehyde in the presence of the immobilized phase-transfer catalyst (PEG 600-PS) was developed. Classic methods of obtaining HPA from IBA and FA use trialkylamine as catalysts [14,15,16,31]. Reactions with their participation take place at higher temperatures (70–100 °C) than the developed method. Another drawback of these conventional approaches is difficulty of separating the catalyst from the reaction mixture. Extraction and/or distillation are required. There is also a known method of HPA synthesis involving potassium carbonate solutions without additional catalyst [13]. However, this method requires the use of high temperatures (65–75 °C) and two-fold molar excess of IBA in relation to FA. Other methods use ion-exchange resins as catalysts [7,17]. The reactions are carried out at a temperature of 40–100 °C and the addition of a solvent, e.g., NPG, in an amount of 10–30 wt.%, is necessary. Kleineberg et al. proposed perovskites and metal nitrides as catalysts for the synthesis of HPA [18]. However, this requires a high temperature reaction (150 °C) and the HPA yields are relatively low (53%). The use of MgO as a catalyst allows one to obtain HPA with high yield, ranging from 95–97%; however, it is necessary to use a high excess of IBA in relation to FA (3–10:1 mol/mol), temperatures in the range of 55–85 °C and a relatively long time (2.5–6 h) [32]. Culp et al. used a base-modified clay catalyst such as LiOH-modified Montmorillonite KSF clay. The advantages of our method in relation to this method are a lower molar ratio of IBA to FA, no additional solvent and a shorter reaction time (2 h instead of 24 h) [33]. Compared to the previously described methods [19,20,21] for the synthesis of HPA with a phase-transfer catalyst, in this work, HPA can be obtained with higher yield and selectivity (IBA conversion >96%, selectivity >98% compared to 76–79% selectivity and 62–72% yield) at a lower temperature and a shorter time (40 °C, 2 h instead of 70 °C, 6–8 h). In comparison to the method presented in [26], the PTC catalyst can be easily recovered by filtration and reused multiple times. Moreover, this method enables easy separation of the product from the reaction mixture using simple phase separation, which can then be used directly for the hydrogenation unit to obtain neopentyl glycol. In addition, the process can be carried out in a continuous mode, which makes it well suited for the chemical industry.

## Figures and Tables

**Table 1:** The effect of IBA:FA molar ratio on hydroxypivaldehyde synthesis. Reaction conditions: 40 wt.% K_2_CO_3_; PEG 600-PS, 0.5 mol%; 40 °C; 600 rpm; 2 h. Each experiment was performed three times.

IBA:FA Molar Ratio	Conversion, %	Selectivity, %
1:1	95	95
1.2:1	96	99
1.3:1	96	98

## Data Availability

Not applicable.

## Supplementary Materials

The following supporting information can be downloaded at: https://www.mdpi.com/article/10.3390/molecules27196459/s1, Figure S1: Syrris Asia flow system equipped with two pumps; Equation S1: Determination of the exact volume of the reactor for HPA synthesis; Figure S2: (a) Fresh and (b) swollen catalyst; Figure S3: NMR spectrum of crude HPA (a) ^1^H (b) ^13^C; Figure S4: GC-MS spectrum of crude HPA; Figure S5: An example of the GC spectrum of reaction mixture during HPA synthesis.
