# Supplementary material for: Polymer-Supported Poly(Ethylene Glycol) as a Phase-Transfer Catalyst for Cross-Aldol Condensation of Isobutyroaldehyde and Formaldehyde
Source: Molecules. 2022 Sep 30;27(19):6459. doi: 10.3390/molecules27196459 (PMC9571435; doi:10.3390/molecules27196459)
Supplement: Supplementary file 1 [file molecules-27-06459-s001.zip › molecules-1900811-supplementary.pdf]

Supplementary Materials

Polymer-supported polyethylene glycol as a phase-transfer catalyst for cross-aldol condensation of isobutyroaldehyde and formaldehyde

Agnieszka Siewniak [1,*], Edyta Monasterska [2,3] , Ewa Pankalla [2] and Anna Chrobok [1]

[1] Department of Chemical Organic Technology and Petrochemistry, Faculty of Chemistry, Silesian University of Technology, Krzywoustego 4, 44-100 Gliwice, Poland
[2] Grupa Azoty Zakłady Azotowe Kędzierzyn, S.A., Mostowa 30A, 47-220 Kędzierzyn-Koźle, Poland;
[3] Department of Chemical Organic Technology and Petrochemistry, PhD School, Silesian University of Technology, Akademicka 2A, 44-100 Gliwice, Poland;
* Correspondence: agnieszka.siewniak@polsl.pl (A.S.)

Figure S1. Syrris Asia flow system equipped with two pumps.

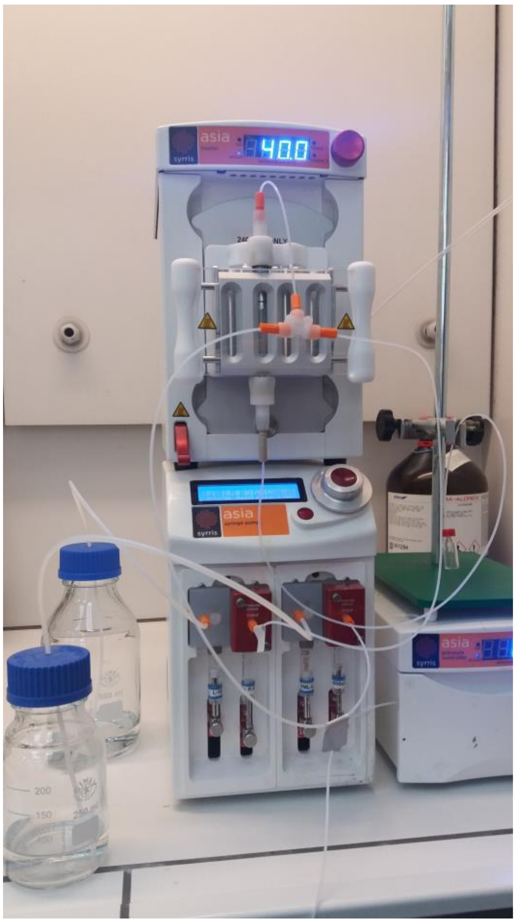

Equation S1. Determination of the exact volume of the reactor for HPA synthesis.
In order to establish the exact volume of the reactor, the reactor with the catalyst was weighed first and then isopropanol was passed through the catalyst bed for 0.5 h at the reaction temperature (40 °C). After swelling, unabsorbed solvent was removed and the reactor swollen catalyst bed was weighed at room temperature.

m_0= mass of reactor with catalyst = 30.75 g
m_1= mass of reactor with catalyst and isopropanol = 31.92 g
Isopropanol = m_1-m_0= 1.17 g
$V=m/d_{isopropanol}$= 1.17/0.78 = 1.5 mL

Figure S2. (a) Fresh and **(b)** swollen catalyst.

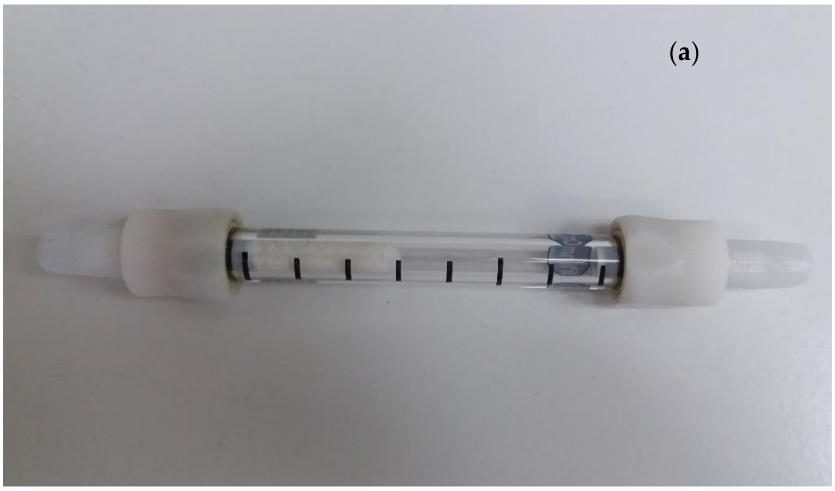

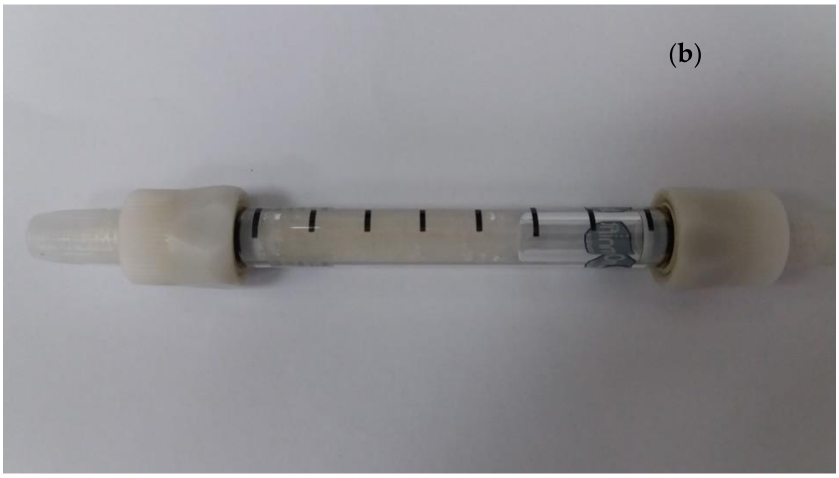

Figure S3. NMR spectrum of crude HPA **(a)** ^{1}H **(b)** ^{13}C.

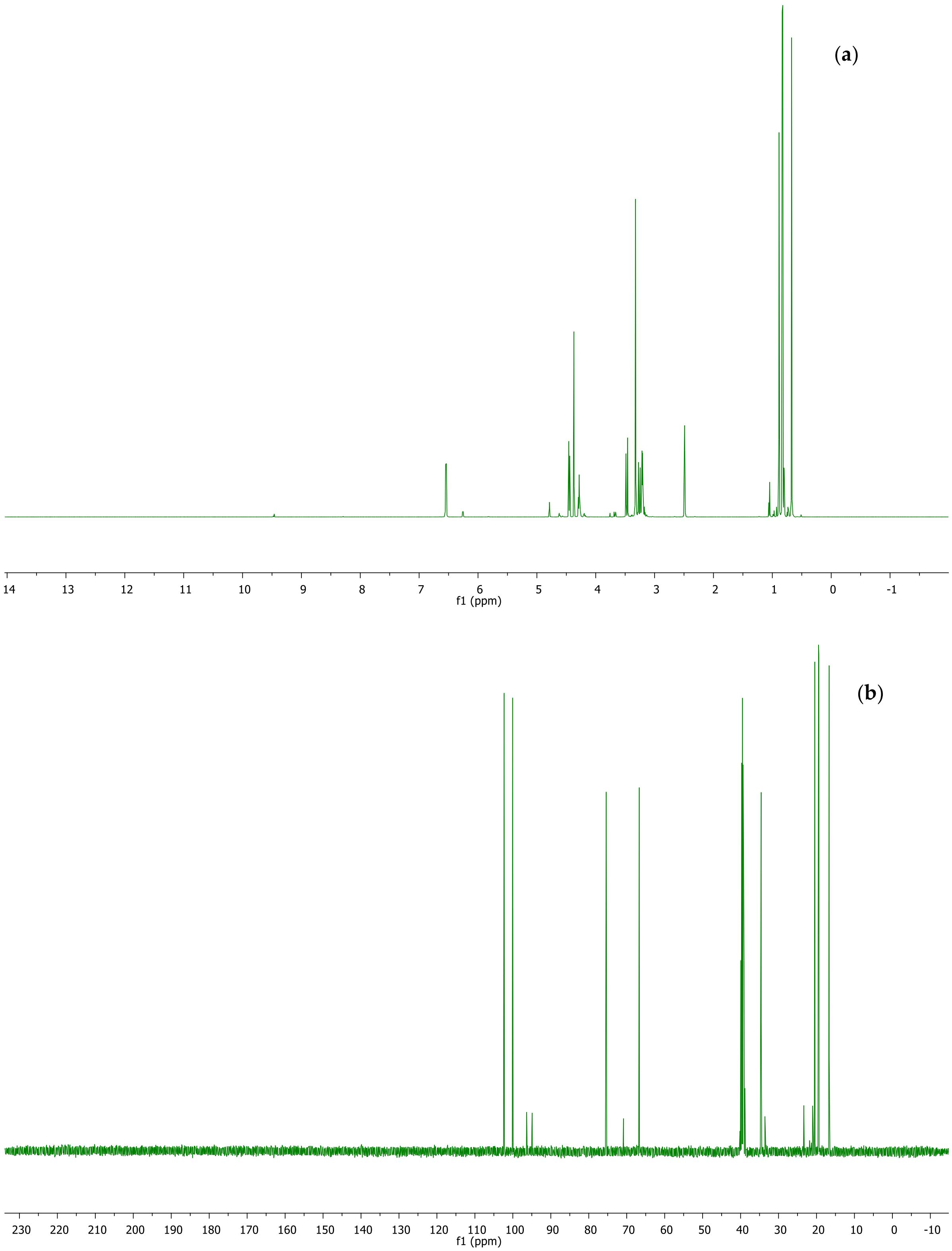

Solvent: DMSO.

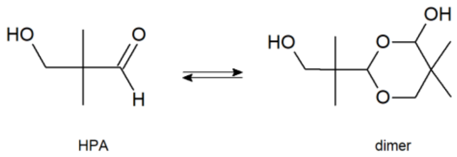

HPA easily dimerizes [1,2]. At higher temperatures, the equilibrium is shifted towards the monomer, and after cooling, towards the dimer [2]. The dimer is a white solid.

Figure S4. GC-MS of crude HPA.

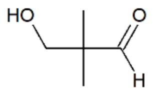

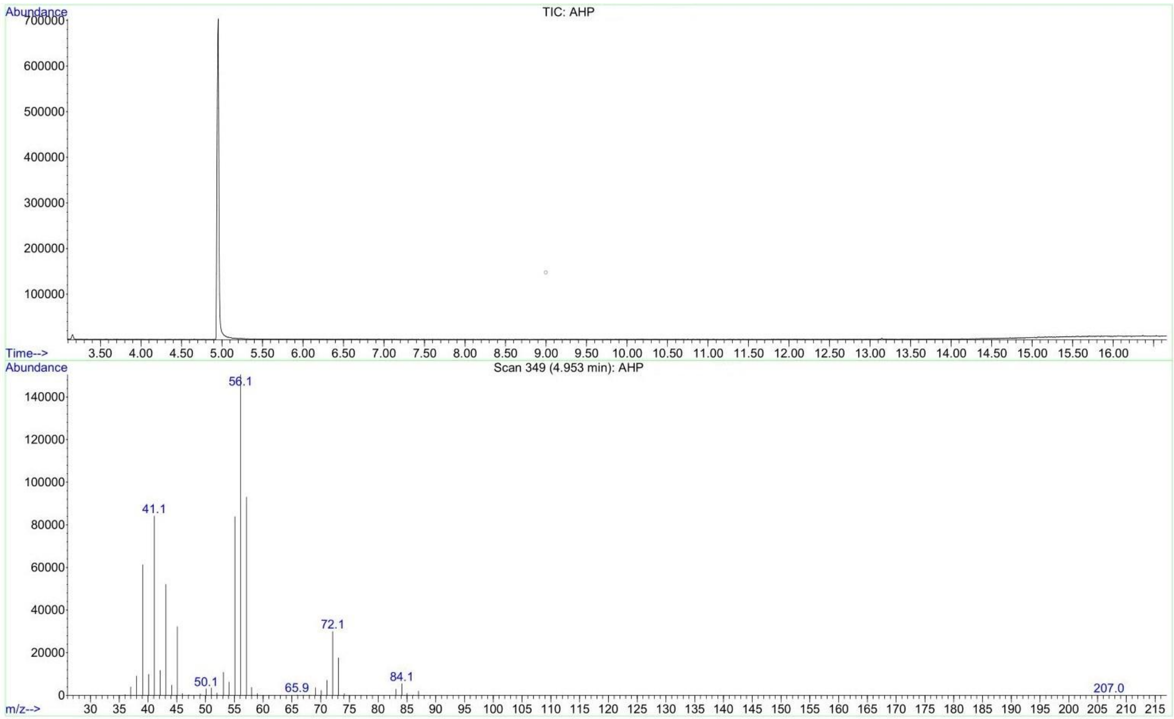

Figure S5. An example of the GC spectrum of reaction mixture during HPA synthesis.

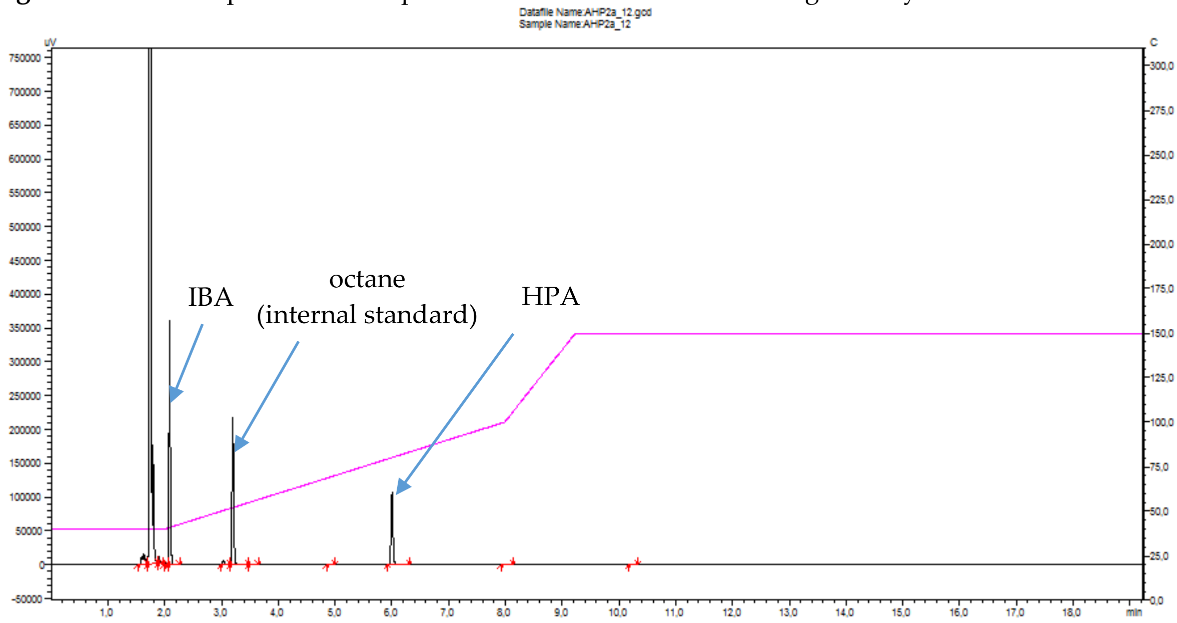

1. Amemiya, J; Watanabe, M.; Kuzuhara, I. Patent EP 1752439, 1 February, 2012.
2. Santoro, E.; Chiavarini, M. *J. Chem. Soc., Perkin Trans. 2* **1978**, 189.
